# Context Is Medicine: Integrating the Exposome into Neurorehabilitation

**DOI:** 10.3390/brainsci15111198

**Published:** 2025-11-07

**Authors:** Rocco Salvatore Calabrò

**Affiliations:** IRCCS Centro Neurolesi Bonino-Pulejo, S.S. 113 Via Palermo, C.da Casazza, 98124 Messina, Italy; roccos.calabro@irccsme.it

**Keywords:** exposome, neurorehabilitation, sleep and circadian rhythms, anticholinergic burden, actigraphy, deprescribing

## Abstract

Neurorehabilitation has become increasingly data-enabled, yet the conditions that most strongly modulate recovery, sleep consolidation, circadian alignment, medication ecology, and social–environmental context are rarely captured or acted upon. This opinion paper argues that an exposome perspective, defined as the cumulative pattern of external and internal exposures and their biological imprints across the life course, is not ancillary to rehabilitation but foundational to making therapy learnable, timely, and equitable. We propose a pragmatic model that centers on a minimal exposure dataset collected in minutes and interpreted at the point of care. Two clinical exemplars illustrate feasibility and utility. First, sleep and circadian rhythms: brief actigraphy and standardized reporting can make daily alertness windows visible, allowing teams to align high-intensity sessions to receptive states and to justify environmental adjustments as clinical interventions. Second, anticholinergic burden: a simple, trackable index can be integrated with functional goals to guide deprescribing and optimize cognitive availability for training. Implementation hinges less on new infrastructure than on workflow design: a short intake that surfaces high-yield exposures; embedding targets, e.g., sleep efficiency thresholds or anticholinergic load reductions, into plans of care; enabling secure import of device data; and training staff to interpret rhythm metrics and burden scores. We outline a parallel research agenda comprising pragmatic trials of bundled, exposure-informed care; longitudinal cohorts with time-stamped exposure streams; and causal methods suited to time-varying confounding, all under explicit equity and ethics safeguards. By measuring a few modifiable exposures and linking them to routine decisions, neurorehabilitation can convert context from a source of unexplained variance into actionable levers that improve outcomes and narrow unjust gaps in recovery.

## 1. Introduction

Neurorehabilitation is steadily evolving from a craft informed by experience into a data-enabled clinical science. We can quantify impairment with exacting precision, map structural and functional lesions with millimetric detail, and meter therapy intensity almost in real time. Yet the determinants that often bend recovery curves after acquired brain injury or neurodegenerative disease habitually lie outside our customary datasets. They reside in “daily ecologies”: ambient noise and light at night, regularity of activity and meals, polypharmacy that blunts attention or postural control, and the social scaffolds that enable or constrain participation. The exposome, the totality of external and internal exposures across the life course and their biological imprints, offers a disciplined way to name, measure, and integrate these influences with the clinical and biological signals we already track. Beyond listing exposures, the framework emphasizes their time-varying, interactive nature and the biological signatures (e.g., inflammatory or autonomic tone) through which they shape readiness to learn. Originally proposed to complement the genome’s explanatory power, the exposome has matured into a practical agenda for health research and, increasingly, translation. What might it add when the clinical endpoint is not merely survival or symptom score but learning, adaptation, and participation? [1,2,3,4]. The case for an exposome lens in neurorehabilitation is mechanistic as much as it is contextual. Plasticity is state-dependent: it is tuned by sleep consolidation and circadian alignment, by inflammatory and metabolic tone, by autonomic balance, and by the cognitive clarity that pharmacology can either support or impair. If synaptic renormalization during sleep underwrites learning, then light at night, nocturnal noise, and irregular ward routines become dose modifiers rather than background features; likewise, the cognitive drag of anticholinergic burden reshapes the same therapy hour’s yield depending on post-dose timing. These are not speculative associations but patterns repeatedly described across basic and clinical science, and they are now feasibly measured with low-burden tools such as actigraphy, brief digital phenotypes, and parsimonious biomarker panels [5,6,7]. The core translational move is to link those measurements to scheduling and dosing decisions at the bedside. Why, then, is the exposome seldom visible in routine pathways? Structurally, documentation captures impairments and delivered units but underspecifies the conditions under which therapy is received; methodologically, most trials omit longitudinal, time-stamped exposures or their interaction with dose and timing; and practically and ethically, teams face heterogeneous measures and privacy concerns without clear action pathways. But these barriers are not insurmountable if we prioritize parsimony, standardization, and actionability from the outset.

Recent reviews chart fast progress in operationalizing the brain exposome across the life course, from neurodevelopment to aging and neurodegeneration. They synthesize how general, specific, and internal exposures interact with neural systems and disease trajectories, reinforcing the translational rationale for exposure-informed rehabilitation [8,9]. Building on this literature, we argue that standardized, low-burden measurements embedded in routine workflows can make the exposome actionable in diverse settings.

The practical value of an exposome lens emerges when measurement changes what a team does today rather than in an abstract future. A narrow set of high-yield signals can be tracked with minimal burden and translated into decisions that clinicians already take, such as when to schedule intensive sessions and how to balance therapeutic efficacy with cognitive availability. This approach treats context as a dose modifier that amplifies or dampens learning from each hour of practice. It also travels well across settings because the same measurements can be repeated during transitions without disrupting care, which supports continuity and enables learning health systems in which outcomes inform iterative refinement of timing and intensity. Demographic variation in rest–activity rhythms underscores why equity must be designed in from the outset. Older adults show lower amplitude and mesor with more advanced acrophase yet greater interdaily stability compared with younger adults, while significant race and sex differences shape baseline patterns (for example, non-Hispanic Asian people exhibit a delayed acrophase with β 0.32 and *p* = 0.001; age-by-race interactions for stability and fragmentation are significant with *p* = 0.04 and *p* = 0.01). Programs should therefore monitor uptake and outcomes across subgroups to ensure that exposure-informed care closes rather than widens gaps [10]. Within this framework, the general external domain captures social and built-environment factors, the specific external domain includes chemicals, infections, behaviors, and medications, and the internal domain reflects physiological states such as inflammation, endocrine tone, and microbiome dynamics [8,9]. For the brain, these domains matter across sensitive windows, during development, after injury, and in later life, when exposures can bias plasticity and recovery trajectories [8,9]. Two exposures are immediately tractable in rehabilitation: sleep circadian physiology and cumulative anticholinergic, sedative load, both linked to attention and learning and to synaptic and systems consolidation mechanisms [11,12,13]. High cumulative anticholinergic burden is associated with faster cognitive decline and greater delirium risk, which can undermine therapy engagement and skill acquisition [14,15]. These links justify a pragmatic capture strategy that uses brief actigraphy blocks for rest–activity rhythms and automated anticholinergic cognitive burden (ACB)/drug burden index (DBI) computation from the electronic medication administration record (eMAR), with results surfaced in the electronic health record (EHR) as simple, interpretable flags that guide therapy timing and deprescribing [16,17]. This approach preserves clinical flow, supports equity through low-cost options, and enables prospective evaluation within learning health systems.

This opinion paper argues that an exposome-aware approach is not ornamental to neurorehabilitation; it is integral to making therapy learnable, timely, and equitable. Our rationale is pragmatic. First, we outline a minimal exposure dataset that can be collected in minutes, interpreted quickly, and linked to specific adjustments in scheduling, dosing, and environment. Second, we illustrate tractable targets, sleep–circadian alignment, and anticholinergic burden, where measurement and action can be embedded in routine care with immediate clinical meaning. Third, we sketch an implementation and research agenda that leverages time-stamped exposure streams, causal designs suited to time-varying confounding, and equity safeguards so that benefits accrue across settings and socioeconomic strata. The objective is straightforward: to convert subtle, modifiable determinants of recovery into visible clinical levers and measurable quality indicators, thereby improving individual outcomes while narrowing unjust variation.

## 2. Exposome-Informed Neurorehabilitation: Concept, Clinical Levers, and Implementation

### 2.1. From Concept to Clinic: A Pragmatic Exposome for Neurorehabilitation

Contemporary neurorehabilitation is highly instrumented, yet whether training consolidates into durable function often hinges on the lived context that shapes brain state at the moment therapy is delivered. The exposome provides a life-course construct that complements genes and images by quantifying cumulative external and internal influences together with their biological traces; crucially, it foregrounds modifiable conditions and their timing, offering a bridge between ecology and mechanism [4]. In practice, the pivotal question is when the nervous system is most receptive and how to align intensive tasks to those receptive windows. Actigraphy-derived rest–activity rhythms correlate with affect and cognition after stroke, suggesting that rhythm-aware scheduling and ward routines could enhance learning efficiency and reduce “state mismatch” between patient and therapy [18]. In parallel, medication ecology is a tractable lever: higher anticholinergic load is associated with cognitive impairment and falls in older adults, yielding a simple, trackable index that can be aligned with functional goals and deprescribing plans during the rehabilitation window [19]. Rather than exhaustive inventories, teams need compact “signals” surfaced alongside goals, sleep regularity, daytime stability, and anticholinergic score, displayed where clinicians make decisions. Many electronic health records already include fields for social determinants of health, enabling linkage between brief exposure screens, therapy dosing decisions, and discharge planning without duplicative documentation [20]. Embedding these fields as active decision prompts (e.g., flags for post-dose cognitive lulls) reduces the gap between measurement and action.

**Figure 1:**
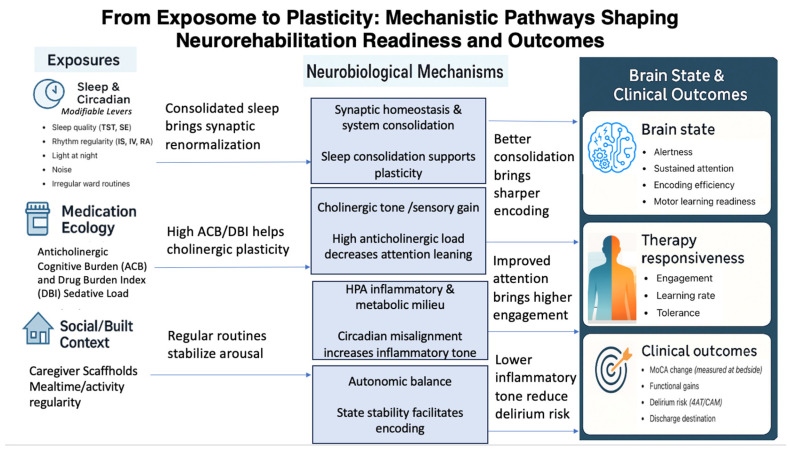
From Exposome to Plasticity: Mechanistic Pathways Shaping Neurorehabilitation Readiness and Outcomes. Abbreviations: TST, total sleep time; SE, sleep efficiency; IS, interdaily stability; IV, intradaily variability; RA, relative amplitude; ACB, Anticholinergic Cognitive Burden; DBI, Drug Burden Index; MoCA, Montreal Cognitive Assessment; 4AT/CAM, delirium screens.

Mechanistic links support clinical relevance. Sleep modulates synaptic renormalization, systems consolidation, and the extraction of statistical regularities; deprivation perturbs molecular signaling and gene expression with downstream effects on plasticity and cognition [10,11,12,21]. Restoring consolidated nocturnal sleep and regular rest–activity rhythms is therefore expected to improve learning efficiency during therapy. Cholinergic tone is a second convergent pathway: acetylcholine regulates attention, sensory gain, and cortical plasticity, and anticholinergic blockade dampens experience-dependent plasticity in humans [18,22]. These pathways justify low-burden measurements that can be mapped to modifiable levers at the bedside. Sleep consolidation supports also synaptic renormalization and systems-level consolidation, which increases the efficiency with which recent experiences are integrated and reduces susceptibility to interference. When circadian rhythms are aligned, attentional fluctuations decrease and the internal milieu becomes more predictable, so patients engage more steadily and tolerate higher cognitive loads during therapy. Anticholinergic exposure pushes in the opposite direction because it reduces sensory gain and impairs encoding; the same schedule can yield less learning under a higher anticholinergic tone. These pathways do not require exhaustive assays to become actionable. They require simple, repeatable signals that indicate whether the brain is ready to learn and whether the pharmacologic background is aligned with the plan of care. Once these signals are visible inside the record, they can be mapped to concrete choices on scheduling and deprescribing, which closes the gap between mechanism and bedside action [23]. Figure 1 summarizes the mechanistic model that links modifiable exposures to neurobiological pathways and, in turn, to brain state, therapy responsiveness, and clinical outcomes. The left-to-right schematic makes explicit how sleep–circadian physiology, anticholinergic/sedative burden, and social/built context converge on synaptic homeostasis and systems consolidation, cholinergic tone, HPA-inflammatory/metabolic milieu, and autonomic balance, mechanisms that shape attention, encoding, and readiness to learn. The figure also annotates bedside measures and decision thresholds to emphasize actionability at the point of care. Bedside screens are logged in parallel (4AT/CAM; MoCA), and the 4AT provides high diagnostic accuracy and is feasible at the bedside. Traffic-light badges and a “high-activity window” flag make the decision logic visible, auditable, and repeatable at the point of care [24].

### 2.2. Actionable Exemplars: Sleep–Circadian Alignment and Anticholinergic Burden

Sleep offers an immediate, tractable target. Consolidated sleep and aligned circadian rhythms support synaptic plasticity and the capacity to learn from therapy; inpatient conditions, nocturnal interruptions, suboptimal light, and noise commonly erode both. Making rhythms visible (sleep efficiency, interdaily stability, intradaily variability) enables chronotherapeutic scheduling of high-load sessions during alertness peaks, while zeitgeber routines (morning light, quiet hours) normalize phase. In this framing, sleep optimization becomes a dose-modifying lever rather than a lifestyle suggestion. Parallel feasibility exists for medication ecology. Polypharmacy and ACB can depress attention, memory, and mobility, diminishing the return on therapeutic effort. Systematic reviews show that anticholinergic burden scales are measurable and associated, albeit with heterogeneity across scales and outcomes, with delirium, cognitive deficits, falls, and mortality in older adults. Stroke rehabilitation studies link higher anticholinergic load with dysphagia, underscoring a direct pathway from medication exposure to functional participation. Tracking a burden score alongside functional targets supports structured deprescribing (substitution, tapering, or dose timing) and coordinates pharmacy-clinician huddles to align pharmacology with session goals. None of this demands perfect evidence; it requires disciplined attention to modifiable exposures during the rehabilitation window [5,19,23,24,25,26,27]. Anticholinergic burden can be applied in practice through a three-step workflow integrated with medication reconciliation. First, quantify exposure at admission and weekly using validated instruments, the ACB scale and the DBI. An ACB score ≥ 3 and a DBI ≥ 1 flag high-risk exposure linked with cognitive and functional harms [28,29,30]. Second, conduct a structured deprescribing review that prioritizes strong antimuscarinics and cumulative sedative load, proposing safer alternatives that preserve therapeutic intent. Third, monitor downstream effects through brief screens for delirium using the 4 ‘A’s Test (4AT) or the Confusion Assessment Method (CAM), and cognition using the Montreal Cognitive Assessment (MoCA), and we track rehabilitation outcomes [31,32]. The 4AT provides high diagnostic accuracy and is feasible at the bedside for routine delirium screening [24]. Cohort data indicate that moderate-to-high cumulative DBI is associated with an additional Mini-Mental State Examination (MMSE) decline of 0.48 and 0.44 points per year compared with no exposure; reducing exposure could plausibly shift this slope favorably [14,15]. Clinicians may also use the International ACB tool to harmonize drug classification at the point of care [33]. Anticholinergic and sedative burden provides a second lever because it is measurable in near real time from the eMAR and modifiable within standard prescribing practices. The Drug Burden Index and Anticholinergic Cognitive Burden summarize cumulative effects in a way that approximates the pharmacologic milieu the brain experiences across the day. These estimates provide clinically meaningful targets for deprescribing programs that aim to improve cognitive availability for training and are consistent with large prospective data showing a graded increase in incident dementia with higher cumulative exposure to strong anticholinergics (HR 1.54; 95% CI 1.21–1.96 for the highest exposure; *p* for trend <0.001) [34]. At the same time, community cohorts of older men indicate that higher DBI is cross-sectionally associated with lower MMSE (coefficient −0.161; 95% CI −0.250 to −0.071) without clear acceleration of decline over five years, a nuance that argues for careful causal modeling and for evaluating both short-term state improvements and longer-term trajectories [35]. In practice, structured deprescribing linked to rehabilitation goals can prioritize substitutions or dose timing that preserve therapeutic intent while reducing anticholinergic tone; downstream effects can be tracked with brief delirium and cognition screens embedded in routine care [23,24]. 7-day actigraphy blocks were used to derive TST, and SE, indices (IS, IV, RA) as standard practice in rhythm profiling [16,36,37,38]. Therapy is then preferentially scheduled within each patient’s stable high-activity window and shifted away from predicted sleep inertia or nocturnal fragmentation. In ambulatory stroke survivors, more regular rest–activity rhythms correlate with better affect and cognition, supporting this alignment while trials test effects on engagement and functional gains [18]. The rhythm features used here translate directly into bedside choices. Total sleep time and sleep efficiency indicate whether the night was restorative, while interdaily stability, intradaily variability, and relative amplitude capture regularity and fragmentation of the 24 h profile. When nights are curtailed or fragmented, patients typically arrive with lower alertness and poorer sustained attention; small environmental changes during the ward routine can shift the state toward readiness within days. A short actigraphy block at admission is usually sufficient to reveal each patient’s high-activity window and can be repeated without disrupting care. Reliability analyses in large older-adult cohorts show that stable estimates of rhythm fragmentation require on average five valid days when validity is defined as at least two-thirds wear in both day and night, with intraclass correlation coefficients reaching the acceptable range; some metrics stabilize earlier, such as transition probabilities in two to three days and intradaily variability around four days. These parameters support feasibility in typical rehabilitation timelines and justify repeating blocks at clinically meaningful intervals [39]. Baseline rest–activity patterns are not uniform across populations, which has implications for individualized scheduling. In a nationally representative sample, older adults had lower amplitude and mesor yet more stable and less fragmented rhythms compared with younger adults, and non-Hispanic Asians exhibited a significantly delayed acrophase relative to non-Hispanic Whites (β 0.32, *p* = 0.001). Interactions between race/ethnicity and age were significant for interdaily stability and intradaily variability (*p* = 0.04 and *p* = 0.01). These differences are not barriers to implementation. They justify monitoring subgroup uptake and outcomes and using diary pathways where wearables are not feasible so that rhythm-aware scheduling can be delivered equitably [10].

### 2.3. Measurement-to-Action, Service Design, and an Evaluative Agenda

**Figure 2:**
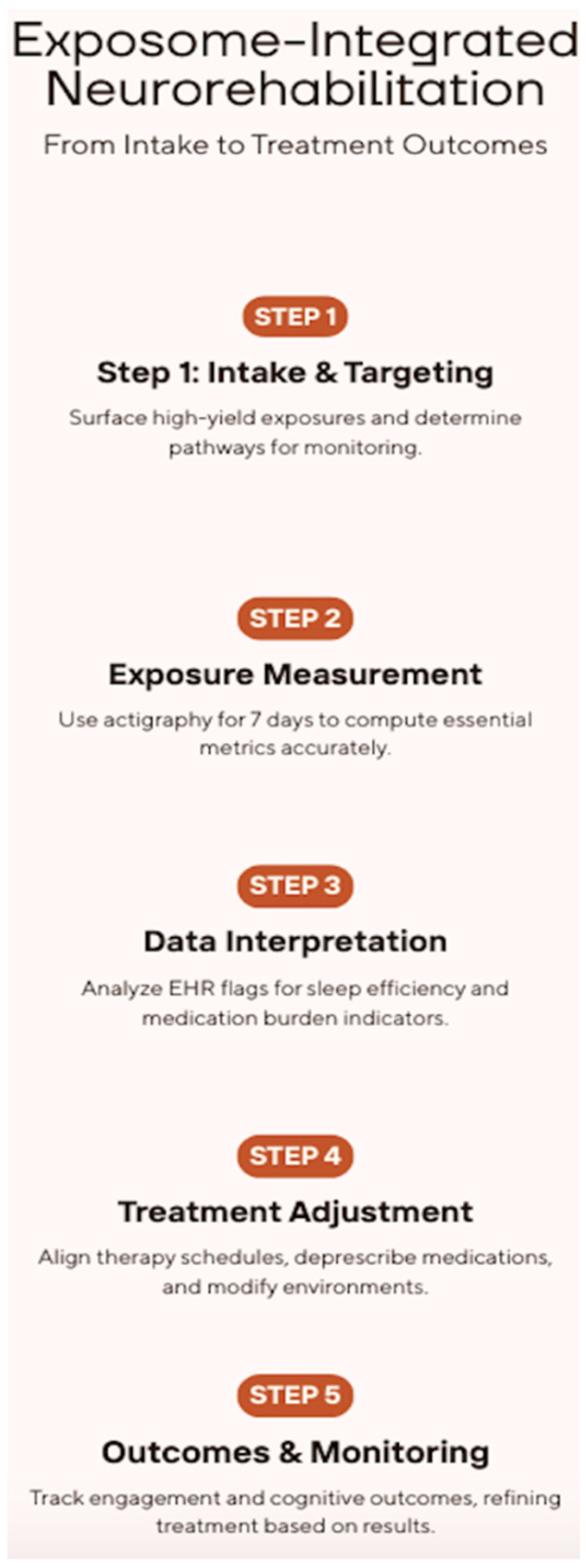
Exposome-Integrated Neurorehabilitation: From Intake to Treatment Adjustment and Outcomes.

A minimal exposure dataset can be standardized across settings without burdening teams. Standardization ensures that teams in different units produce comparable outputs and can learn from pooled experience. A seven-day actigraphy block at admission can be repeated at milestones without disrupting care, and processing with consistent algorithms yields device-agnostic metrics. Reliability work indicates that five valid days, each with at least two-thirds wear during day and night, are sufficient for stable estimates of interdaily stability and activity balance, while intradaily variability typically stabilizes around four valid days; transition probabilities may stabilize with two to three days. These thresholds can be embedded in analytic pipelines so that clinicians always know when data are interpretable [39]. Medication data are already structured and can be transformed into ACB and DBI automatically each day, with pragmatic thresholds such as ACB three or more and DBI one or more prompting review without generating alert fatigue. Therapy encounters recorded with time stamps complete the chain by linking exposures to the sessions where learning occurs, which allows services to test whether rhythm-aware scheduling and deprescribing are followed by measurable improvements in same-day performance [24,35,37,40]. For sleep circadian dynamics, collect total sleep time, sleep efficiency, interdaily stability, intradaily variability, and relative amplitude from wrist actigraphy at baseline and in 7-day blocks monthly, with optional sleep diaries when wearables are not feasible [16,36]. For anticholinergic exposure, extract medication names and doses from the eMAR to compute ACB and DBI daily, with weekly multidisciplinary review for deprescribing [28,29,30]. Time-stamp therapy encounters and record same-day task performance alongside brief delirium and attention screens during acute phases [31]. This dataset links exposures to proximal neurobehavioral states and to functional gains, enabling learning health systems to refine timing and intensity over successive cycles. Electronic records should surface these measurements where decisions are made. Summaries of sleep and rhythms can appear in views used for scheduling so that clinicians align higher cognitive loads with periods of greater alertness, while medication burden appears next to the plan of care with stable thresholds that suggest review rather than alarm. Figure 2 translates these measurement outputs into a six-stage, role-based workflow, from intake and exposure measurement to EHR-embedded interpretation, treatment adjustment, and outcomes with a feedback loop. Two decision nodes define routine checks, sleep efficiency below target and ACB ≥ 3 or DBI ≥ 1, triggering, respectively, rhythm-aware scheduling and a pharmacist clinician deprescribing huddle.

Feasibility has been demonstrated in a prospective study in radiotherapy where biometric data from consumer wearables were integrated into the EMR, with physician review achieved in 95% of participants against a predefined 90% threshold. Patient-reported usability was high with a median System Usability Scale score of 82.5 (IQR 70–87.5). Over longer radiation courses, exercise minutes decreased (*p* = 0.01) and mean daily heart rate variability increased (*p* = 0.02), indicating that the pipeline can track physiologic change over time while fitting routine documentation [41]. Training can therefore concentrate on interpretation and action mapping rather than on device minutiae, which lowers barriers to adoption and keeps attention on patient-facing changes. Furthermore, evaluation should combine pragmatic trials and causal analyses embedded in routine care. A stepped-wedge cluster design lets each unit function as its own control while ensuring that all sites ultimately adopt the bundle. Roll-out can follow operationally stable periods, and fidelity is monitored by verifying that exposure measurements are present and that scheduling and deprescribing decisions refer to those signals. Primary outcomes should include functional gains and discharge destination, while secondary outcomes such as delirium incidence, change in MoCA, therapy tolerance, gait speed, and length of stay provide mechanistic and operational context. Mixed-effects models estimate differences while accounting for clustering, and marginal structural models address time-varying confounding introduced when teams act on measured exposures. Directed acyclic graphs clarify required adjustments, and negative controls help detect residual bias. The clinical relevance of reducing delirium risk and burden is well documented because delirium is consistently associated with longer length of stay and higher mortality; in hospital-wide analyses the presence of delirium increased length of stay by several days and meta-analyses show elevated mortality with pooled odds ratios in the range of 1.7. These benchmarks justify tracking delirium as a safety signal in exposure-informed care [42,43]. A stepped-wedge cluster design across rehabilitation units can also compare usual care with exposome-informed scheduling and deprescribing, enrolling adults after stroke or acquired brain injury who can wear actigraphy or provide proxy data [36,44,45]. Primary outcomes could include functional independence gains and discharge destination; secondary outcomes could include delirium incidence, MoCA change, gait speed, therapy tolerance, and length of stay [32]. Mixed-effects models estimate between-arm differences, while marginal structural models address time-varying confounding from changing sleep and medication exposure [46,47]. Prespecified directed acyclic graphs, negative control exposures, and sensitivity analyses strengthen causal interpretation.

Measurement should remain simple enough to sustain and meaningful enough to steer decisions. Actigraphy during the inpatient phase and early weeks post-discharge yields objective, time-stamped data on rhythms and sleep; brief patient-reported measures can capture fatigue, stress, and barriers to participation; and a small set of environmental or social indicators can be retrieved from existing datasets or community partnerships without duplicative documentation. Crucially, signals must change decisions: session timing anchored to alertness windows; caregiver-negotiated home plans that reflect commute and noise constraints; and explicit reassessment of drugs that compromise attention. Emerging work in stroke populations links rest–activity rhythm features to affective and cognitive outcomes and to functional gains during rehabilitation, reinforcing the clinical utility of these digital biomarkers as guideposts for dosing and scheduling therapy. At the service level, programs can embed a minimal exposure dataset into the electronic record, enable secure import of device data, and train staff to interpret rhythm metrics and medication burden scores; managers can monitor exposure-sensitive indicators, time to sleep normalization, reduction in anticholinergic burden, adherence contextualized by home constraints, and use them to drive quality improvement. Evaluation should use pragmatic, multi-site designs (including stepped-wedge rollouts), statistical process control for run-time feedback, and causal methods that account for time-varying confounding; mediation analyses can link exposures to biological intermediates and then to clinical endpoints. Equity and ethics must be explicit privacy-by-design, proportionate consent, role-based access, non-pathologizing language, and support pathways for patients facing structural constraints. Policymakers and payers can accelerate uptake by incentivizing a small number of exposure-sensitive quality metrics and funding community partnerships that enable practical changes after discharge [48,49].

Illustrative expectations help calibrate implementation. Patients who move from high to lower cumulative DBI could plausibly see a reduction in the annual rate of MMSE decline on the order of half a point, in line with cohort estimates for moderate-to-high exposure [14,15]. Stabilizing rest–activity rhythms and improving sleep efficiency are associated with better affect and cognition after stroke; aligning therapy with each person’s high-activity window is expected to improve engagement and learning curves, which ongoing pragmatic trials can test [18,36].

Implementation faces predictable barriers. Data governance must address privacy and consent for patient-generated health data with general data protection regulation aligned policies that favor data minimization, on-device preprocessing, and de-identification before analytics [17]. EHR integration requires reliable ingestion of actigraphy summaries and medication data; templated flowsheets and standardized order sets reduce variability, and small-scale pilots show feasibility of real-time wearable to eMAR pipelines [50]. Training should focus on pragmatic interpretation rather than device minutiae. Reimbursement can be supported by linking exposure management to safety and functional outcomes. Accessibility demands low-cost options such as device-loan libraries, fallback paper sleep logs, and medication reviews decoupled from wearables to avoid digital exclusion. Privacy and equity are design constraints rather than afterthoughts. Patient-generated data should be collected with proportionate consent that explains what is measured, how it is interpreted, and how it informs care. Data minimization and de-identification before analytics protect patients while allowing services to learn from their own data. Access to the benefits of exposure-informed care must not depend on personal resources. Device-loan programs and bring-your-own-device options reduce barriers, and diary-only routes preserve the core scheduling signal when wearables are not feasible. Monitoring of uptake and outcomes across socioeconomic strata and demographic groups is essential, supported by nationally representative and cohort data showing age-, sex-, and race/ethnicity-related differences in IS, IV, RA, and timing, including 1 h earlier activity midpoints in mid-to-older adults versus 18–29 years (β −1.0 h; 95% CI −1.6, −0.4) Equity requires verifying that improvements are shared rather than concentrated [51].

## 3. Conclusions

An exposome-aware approach reframes neurorehabilitation as the orchestration of learning under modifiable conditions. By naming and measuring the exposures that tune plasticity, sleep consolidation, circadian alignment, medication ecology, and environmental and social constraints, we move from explaining variance after the fact to actively reducing it. The clinical logic is direct: if therapy depends on attention, alertness, and the capacity to consolidate gains, then interventions that strengthen these preconditions are not ancillary but intrinsic to dose, timing, and sequence. The operational logic is equally practical: a minimal exposure dataset can be collected in minutes, embedded in existing records, and linked to a small set of actions that teams can own, aligning therapy with observable rhythms, instituting sleep-conducive ward routines, quantifying and reducing anticholinergic burden when safe, and negotiating home constraints explicitly in discharge planning. Sustained progress will rely on pairing implementation with evaluation. Time-stamped exposure streams allow us to test whether synchronizing high-intensity sessions to stable daytime activity improves functional independence, whether deprescribing that lowers anticholinergic burden by a defined margin augments therapy responsiveness, and whether early normalization of rest–activity rhythms predicts fewer complications and readmissions. Pragmatic trials and adaptive designs can answer these questions without delaying uptake. Throughout, equity remains a central criterion of success: exposure-informed care must benefit patients across socioeconomic strata, not just those with access to devices or quiet homes. Neurorehabilitation’s comparative advantage is its orientation toward behavior change and participation. By embracing the exposome, the field can broaden what counts as clinically relevant data without losing parsimony, focus on what teams can act upon today, and evaluate iteratively. The goal is not comprehensive surveillance; it is disciplined measurement of a few high-yield exposures, linked to routine decisions that patients can feel and clinicians can deliver. Parsimony is the mechanism that turns a complex construct into a practical tool. The same measurements that guide scheduling and deprescribing can be repeated as patients transition across settings, which supports continuity and guards against loss of progress. Because signals are embedded in the record and mapped to explicit actions, services can scale the approach and refine it as outcome data accrue. If pragmatic trials confirm gains in function and reductions in delirium and length of stay, exposure-informed care can be implemented as a safety and quality bundle across rehabilitation units that differ in resources and patient mix [41]. In doing so, we turn context from an afterthought into a lever-one that measurably improves outcomes and narrows unjust gaps in recovery.

Exposome-informed neurorehabilitation could be feasible with a minimal dataset that captures sleep and circadian physiology and anticholinergic burden, links these to therapy timing and deprescribing, and closes the loop with functional outcomes. Mechanistic evidence indicates that consolidated sleep supports plasticity, while excessive anticholinergic load impairs attention and learning [6,7,8,9]. Embedding these levers into daily workflows offers a credible path to safer care and better recovery, and positions pragmatic trials to test real-world impact [18,19,20]. This approach translates two modifiable levers into routine practice through interpretable EHR flags for circadian alignment and deprescribing triggers at ACB ≥ 3 or DBI ≥ 1. Reducing cumulative sedative anticholinergic load is expected to slow cognitive decline and to lower delirium risk, which protects learning during rehabilitation. Stabilizing rest–activity rhythms is expected to increase practice-dependent plasticity and engagement, consistent with observed links between rhythm regularity, affect, and cognition after stroke. Near-term benefits are measurable through 4AT or CAM for delirium, MoCA for cognition, and unit-specific performance and functional metrics. Validation is tractable via stepped-wedge cluster trials embedded in services and by causal analyses that address time-varying sleep and medication exposure. The model also supports equity by enabling device-loan actigraphy or diary-only pathways and by decoupling medication review from continuous wearables. If confirmed in pragmatic studies, exposure-informed care can be scaled as a quality-and-safety bundle for neurorehabilitation.

## Data Availability

No new data were created or analyzed in this study. Data sharing is not applicable to this article.
